# Introducing Telemedicine in Italy: Citizens’ Awareness of a New Healthcare Resource

**DOI:** 10.3390/healthcare11152157

**Published:** 2023-07-28

**Authors:** Francesca Gallè, Stefania Oliva, Edoardo Covelli, Antonio Del Casale, Giovanna Da Molin, Giorgio Liguori, Giovanni Battista Orsi, Christian Napoli

**Affiliations:** 1 Department of Movement Sciences and Wellbeing, University of Naples “Parthenope”, Via Medina 40, 80133 Naples, Italy; giorgio.liguori@uniparthenope.it; 2 Department of Medical Surgical Sciences and Translational Medicine, Sant’Andrea University Hospital, Sapienza University of Rome, Via di Grottarossa 1035/1039, 00189 Rome, Italy; stefania.oliva@uniroma1.it (S.O.); christian.napoli@uniroma1.it (C.N.); 3 Department of Neuroscience, Mental Health and Sense Organs (NEMOS), Sant′ Andrea University Hospital, Sapienza University of Rome, Via di Grottarossa 1035/1039, 00189 Rome, Italy; edoardo.covelli@uniroma1.it; 4 Department of Dynamic and Clinical Psychology and Health Studies, Faculty of Medicine and Psychology, Sapienza University, Via di Grottarossa 1035/1039, 00189 Rome, Italy; antonio.delcasale@uniroma1.it; 5 Inter-University Research Centre “Population, Environment and Health”, University of Bari Aldo Moro, Piazza Cesare Battisti 1, 70121 Bari, Italy; giovanna.damolin@uniba.it; 6 Department of Public Health and Infectious Diseases, “Sapienza” University of Rome, Piazzale Aldo Moro 5, 00185 Rome, Italy; giovanni.orsi@uniroma1.it

**Keywords:** telemedicine, delivery of healthcare, information technology

## Abstract

In recent years, especially during the COVID-19 pandemic, new technologies have emerged as useful resources in healthcare. Telemedicine services may decrease opportunities for contagion by limiting direct medical contacts; they can lead to greater access to and better quality of care, especially for the elderly and chronically ill patients. However, there are still some difficulties in their widespread use, such as lack of knowledge about the effectiveness and safety of telemedicine; lack of awareness of its existence; privacy issues; and lack of computer literacy. The aim of this study is to assess the awareness of and attitude toward telemedicine in the Italian adult population, considering sociodemographic characteristics and territorial differences in telemedicine service implementation. A questionnaire was administered to Italian citizens from October 2022 to February 2023 using communication and social media in order to collect sociodemographic and health characteristics and data on awareness and use of telemedicine services. Less than half of the respondents (*n* = 1002) were aware of telemedicine services in their region; most of them did not use the available services due to a preference for in-person visits or lack of need. More than 90% of participants who used these services were satisfied with them. A negative attitude toward telemedicine was found in a higher proportion of older adults. This study demonstrated that, although telemedicine services are active in Italy, a large part of the population ignores its availability. Therefore, further efforts should be made to increase citizens’ awareness and the use of telemedicine in our country.

## 1. Introduction

In the last decade, especially in light of the COVID-19 pandemic, improvements in information technology have led to increased accessibility and quality of digitally delivered healthcare services [1,2,3].

In this context, the new terms telehealth and telemedicine have been adopted to indicate the application of the new technologies to healthcare services [2,3]. Telehealth refers to the use of telecommunications and information technology in healthcare delivery and patients’ information and education. Within the wider concept of telehealth, telemedicine refers specifically to the use of new technologies for clinical services, such as video consultations, the transmission of clinical data, medical education, remote patient monitoring, and applications for wireless health devices [2,3]. The new technologies, especially via the Internet, allow both patients and providers to store and consult medical information in electronic medical records and to record and transmit data through cameras, digital measurement devices, and wearable biosensors; in addition, patients can meet physicians via live video in real time [2,3]. Therefore, telemedicine may increase access to care for remote places, especially for individuals living in rural areas, and decrease healthcare costs. Moreover, the new technologies can allow teleconsultation between doctors, which can lead to many benefits for patients’ health, such as quick diagnoses and prompt access to adequate treatments [4,5].

Since telemedicine does not imply direct contact between healthcare personnel and other patients, its use has been reported as crucial during health emergencies related to infectious diseases, such as COVID-19 [6,7,8]. Even though telemedicine has known impressive growth in the course of the COVID-19 pandemic, several factors limit its spread. Among these, in addition to the difficulties of performing some types of remote physical examinations, which concern some medical specialties, the lack of knowledge about the efficacy and safety of telemedicine, about the availability of telemedicine services, or about how to access telemedicine visits, together with patients’ preferences toward different providers, have been identified as barriers to telemedicine [9,10]. In the U.S., a study performed to assess telehealth satisfaction showed that nearly three-quarters of consumers were unaware of telemedicine services [11]. The most frequently reported patient barriers to telemedicine use were age, level of education, computer literacy, unawareness of services, and bandwidth [10]. In addition, it should be considered that those who were older, lived in rural areas, had less education, and had more chronic conditions were less likely to have access to the internet than their counterparts [12]. Furthermore, since sharing health information online poses privacy issues, patients who are not able to understand or mistrust privacy policies are often reluctant to use telemedicine services [2,10]. In Italy, the first national guidelines on telemedicine were approved by the General Assembly of the Superior Health Council in 2012 [13]. Since that year, several actions have been taken nationwide to promote the adoption of telemedicine, mainly in light of the COVID-19 pandemic. In 2015, a regulation on electronic health records was issued (Decree of the President of the Council of Ministers 29 September 2015, n. 178). Finally, the document “National guidelines for the provision of telemedicine services”, approved in 2020, provided indications for the provision of some telemedicine services, such as televisits, medical health teleconsultations, teleassistance by health professionals, and telereporting [13].

Since 2012, all the Italian regions have progressively implemented the first national guidelines with their own resolutions, and many differences exist in telemedicine provision throughout the Italian territory. The national mapping performed in 2018 by the Telemedicine Working Group of the New Health Information System found notable discrepancies between regions, with the numbers of experienced telemedicine services ranging from 1 to 36 [14].

This study aims to assess the awareness and the attitude toward telemedicine in the Italian adult population, with regard to sociodemographic characteristics and territorial differences in telemedicine service provisioning.

## 2. Materials and Methods

This cross-sectional study was performed in Italy during the period of October 2022–February 2023 through the use of a Google form. Participants were asked to provide their informed consent before accessing the questionnaire. The Scientific and Ethical Board of the Inter-University Research Center “Population, environment and health” (CIRPAS) approved the study (approval n. 1810_2022).

### 2.1. Participants

The questionnaire was disseminated using communication media (i.e., mailing lists and instant messaging applications) and social networks (i.e., Facebook and Instagram), asking participants to further spread the questionnaire among their contacts. Inclusion criteria were being Italian and adult. Considering a response proportion of 50%, a 95% confidence level and a 5% margin error, it was calculated that a minimum sample size of at least 385 individuals was needed. In order to increase the representativeness of the sample, we accepted all questionnaires obtained within the stated period of the study.

### 2.2. Questionnaire

The electronic questionnaire included three sections (Supplementary Materials). The first aimed to collect the sociodemographic, behavioral, and health-related characteristics of participants. In particular, they were asked to report their sex, age, region of residence, educational level, occupational status (student/unemployed/worker/retired), sentimental/marital status (married/cohabiting or not with a partner/separated or divorced/widowed/single), residential status (living in their own home/other’s home/nursing home), whether they had children, and current health chronic conditions. The second and third parts focused on the participants’ knowledge and use of telemedicine services (e.g., televisits, medical video consultations, and telemonitoring) and electronic health data records.

The questionnaire was tested in a pilot study involving twenty people (data not published). Four experts in public health, epidemiology, and communication technologies designed the questionnaire based on the scientific literature, the aim of the study, and the current status of telemedicine in the country. Subsequently, an external panel of experts evaluated the tool to evaluate its content validity. Furthermore, the questionnaire’s comprehensibility was evaluated in the pilot group. Participants were asked to rate each question on a 7-point scale, with 1 corresponding to “not meaningful” and 7 to “very meaningful”; a mean score of 5 per question was considered as the cut-off for acceptability. To this aim, 10 additional questions reporting grammatical and semantic errors were included in the original questionnaire to assess answer variability. The original questions showed a mean score >5 each, while the additional questions had a mean score <2, confirming the clarity of questionnaire content.

The reliability of the final version of the questionnaire was assessed using Cronbach’s alpha as a coefficient of internal consistency reliability. The questionnaire achieved an alpha value of 0.75 during the pilot study and an alpha value of 0.74 during the larger study, demonstrating satisfactory internal consistency [15].

### 2.3. Statistical Analysis

A descriptive analysis was carried out on the sociodemographic and health characteristics of participants. Continuous variables were expressed as mean values ± standard deviations (SDs), while categorical variables were expressed as numbers and percentages (%) of respondents for each category. Respondents were then categorized into two groups based on their attitudes toward using telemedicine or electronic health data records (willing to use indicating positive attitude and unwilling to use indicating negative attitude). The sociodemographic and health characteristics of the participants were compared between the two groups using a chi-squared test with Yates correction. Those characteristics that significantly differed between the attitude groups were included in multinomial logistic regression models, which were performed considering negative attitude toward telemedicine and electronic health data records as dependent variables. Specifically, a value = 1 was assigned to a positive attitude toward telemedicine/health data records, while a value = 0 was assigned to a negative attitude. Age (expressed as 0 = lower or equal/1 = higher than the median value), sex (0 = male/1 = female), having a sentimental relationship (0 = non-engaged/1 = engaged), being a parent (0 = no/1 = yes), educational level (0 = non-graduated/1 = graduated), employment status (0 = unemployed/1 = employed), and having a concurrent health condition or disease (0 = no/1 = yes) were included in the regression analysis as independent variables. The results were expressed as odds ratios (ORs) and 95% confidence intervals (95% CIs). A *p*-value < 0.05 was considered statistically significant. Statistical analyses were conducted using SPSS (Statistical Package for Social Science; version 28.0; IMB SPSS; Armonk, NY, USA).

## 3. Results

A total of 1002 complete questionnaires were collected. Table 1 shows the sociodemographic characteristics of the sample.

The sample had a wide age range and showed quite equal distributions in the categories of sex, educational level, parent status, and chronic condition. The gender and age characteristics of the sample were quite similar to those of the general Italian population of the same age range (female gender: 51.6%; mean age: 51.9 years), as deduced by the data published by the Italian Institute of Statistics for the year 2023 (http://dati.istat.it/ (accessed on 30 June 2023). The majority of participants came from the center of Italy, were married, were employed, and lived in their own houses.

Table 2 and Table 3 show the answers given by participants to the questions regarding telemedicine services and electronic health data records.

Less than half of the respondents were aware of telemedicine services in their region, while the majority of them were not sure about the activation of electronic health data records. Doctors were reported as the main source of information in both cases. The majority of the participants did not use the available services, mainly because of a preference for traditional visits or lack of need. Among those who preferred in-person services, the lack of trust in telemedicine effectiveness or functioning was the main reason reported. However, more than 90% of those who used the new services were satisfied with them, and a similar proportion of those who did not use telemedicine due to its unavailability were willing to use it.

As for the attitude toward telemedicine, Table 4 and Table 5 show the results of the chi-squared tests performed comparing the sociodemographic characteristics of participants who were favorable and unfavorable toward the use of telemedicine services and electronic health data records.

The group with a negative attitude toward telemedicine showed a significantly higher proportion of individuals with higher age than the other group.

The group with a negative attitude toward the use of electronic health data records showed significantly higher proportions of females, parents, individuals coming from the south of Italy, unemployed, and individuals not living in their own houses with respect to the group with a positive attitude.

In the regression analysis, age was found to be inversely related to a positive attitude toward telemedicine services (OR = 0.432, 95% CI: 0.238–0.784, *p* = 0.006); a positive attitude toward the use of electronic health data records was positively related with female gender (OR = 2.291, 95% CI: 1.759–2.985, *p* < 0.001).

## 4. Discussion

This study provides a picture of the current awareness and attitudes toward telemedicine among Italian citizens. The findings show that less than half of the sample was aware of the available telemedicine services, and less than 40% used them.

Although these findings are in line with the previous literature [11], it seems very surprising since the recent pandemic has determined an unprecedented recourse to new technologies for social and work activities, even among older adults [16,17,18,19].

Furthermore, among those who did not use the available services, the majority preferred traditional visits to televisits or declared their lack of need for electronic health data records. This appears to be in line with previous evidence from the same population, showing that Italian people preferred in-person relationships and sociocultural activities, even during the pandemic [20].

However, our results show that almost all the telemedicine users were satisfied with the accessed services. Furthermore, the majority of the respondents who were not aware of the telehealth services available in their region declared their willingness to use telemedicine.

Considering the possible benefits of telehealth services for patients, healthcare professionals, and the environment, this allows hope for a widespread diffusion and acceptance of telemedicine in the Italian territory.

In our sample, doctors emerged as the reference figures for information in this field. This is an encouraging result since a sample of Italian physicians involved in a pre-COVID-19 survey showed generally low levels of engagement in telemedicine and lower intention to use telemedicine among doctors of higher seniority, indicating a possible reluctance to change practicing habits [21]. Allowing a greater knowledge of patients and their needs, telemedicine and televisits may enable healthcare workers to better manage visiting and travel times and, consequently, enhance performance and thereby reduce work-related stress [2]. Considering the influence that doctors’ indications may have on patients’ choices [22], healthcare professionals should be aware of these potential benefits to increase confidence in telemedicine.

In our study, in accordance with current scientific evidence, a negative attitude toward telemedicine was associated with older age [10,23]. The elderly may experience greater difficulties in accessing and using digital health technologies. Many other barriers to technology use for older people include lower levels of digital literacy, lack of perceived usefulness, lack of access to and experience and skills with digital tools, and physical (especially visual impairments) and cognitive deficits that may hinder the use of these tools. This confirms previous findings regarding the use of digital resources to exercise during the pandemic in the Italian population [20].

With reference to this, it should be considered that elderly patients would be the most likely category to benefit from telemedicine and telehealth services. In particular, they could benefit from telemedicine thanks to the possibility of being visited through videoconferences, eliminating inconveniences related to travel costs and times, waiting times, and reduced autonomy. As a matter of fact, in a U.S. investigation, high proportions of unaware customers were found among participants living in rural or suburban areas, and those who indicated their health as “poor” had never used telemedicine, which shows that patients who may benefit the most from its services use it the least [11].

Furthermore, in our sample a positive attitude toward electronic health data records was related with female gender. This is in line with the higher adoption and satisfaction rates registered among female participants in other surveys [11,24,25].

Interestingly, in line with the previous literature [11], having a chronic condition, which may require periodical medical consultations and can benefit from telemedicine services, was not found to be related with the willingness to use them. Our previous study showed that having a chronic condition was inversely related with the use of web resources, as well as social and cultural activities and maintaining relationships [20], which may lead to social isolation in an emergency situation. At the same time, social isolation is associated with having more chronic diseases, generating a vicious circle [26].

On the other hand, the implementation of telemedicine services is not easy in terms of resources needed and patient acceptability. As for the resources, it should be considered that project costs are high: when a healthcare organization or a team of doctors and programmers decides to implement an effective telemedicine project (monitoring of elderly patients, remote evaluation of a disease, second opinion, etc.), it is necessary to deal with feasibility studies and technical economic evaluations. In every teleconsultation project, a very considerable organizational effort is required between doctors, computer scientists, and programmers to design the necessary software to start the pilot phase. A versatile technological infrastructure that is continuously updated by technical staff; the latest generation of secure servers certified with uninterruptible power supplies and back-ups so as not to lose data; easy and intuitive data and image entry and management procedures with automated control systems and alerts for inappropriate data entry; fast downloading and uploading of high-pixel-density images with zoom and image comparison software; different access profiles for customized data visualizations; and applications for use of the procedures from mobile devices are required. Furthermore, efforts are needed to channel projects into services available for the whole population.

Finally, the market may not yet be ready to welcome the multiple and different telemedicine projects, and difficulties can be encountered when integrating telemedicine with existing care-delivering systems. Above all, the regulations are not yet unified, and government guidelines and sustainability guidelines have only recently been given in some countries, such as Italy. This leads also to the offering of different services throughout a country: currently, some Italian regions provide to citizens electronic data records, and others still do not; even in the same region, some healthcare institutions offer remote visits and consultations for some medical specialties, and others still do not. Furthermore, from the perspective of healthcare mobility, the use of shared software in medical informatics for teleconsultations is still difficult terrain, both for the usability of data software and for image transfer and sharing [27,28]. These aspects can also contribute to creating a negative patient attitude toward telemedicine. Security and privacy aspects are also important to increase acceptability and must comply with new regulations regarding data protection and encryption, especially for web-based platforms, such as with bureaucratic and legal procedures, informed consents, and codes of conduct for doctors [28].

Due to these multiple technical and human factors implicated, participation, responsibility, and desire for effective collaboration among all the stakeholders are the necessary requisites for success in telemedicine adoption. A suitable “network” culture with evaluation of accesses, acceptability, quality of the transmitted data, and medical efficacy should be created for this aim. Furthermore, in order to increase confidence in telemedicine, it would be useful if telemedicine were taught today in health degree courses [29,30].

This study has some limitations. First of all, the recruitment procedure did not allow us to obtain a nationwide representative sample; therefore, our results are not extendable to the whole Italian population. Moreover, the use of an electronic questionnaire and the recruitment procedure allowed us to involve in the study people who use social media and the Internet, who are probably more keen to use digital health than those who do not. Due to its nature, the study was based on subjective thoughts and beliefs, which may not reflect the attitudes of the whole Italian population toward telemedicine. Furthermore, the lack of an updated database of telemedicine services provided in the whole Italian territory prevented an in-depth analysis of the participants’ awareness at a regional level. In addition, in order to avoid excessive length of the questionnaire, the study was not aimed at analyzing the specific types of telemedicine service used by participants, which hindered the detection of possible correlations with their characteristics or health conditions. This aspect can help to better characterize the satisfaction toward telemedicine and should be addressed in further studies.

However, to the best of authors’ knowledge, this is the first study that analyzed the process of telemedicine implementation in Italy from the citizens’ point of view. Since patient perspective is crucial for integrating new technologies into healthcare practice [31], these findings can be useful to address this process.

## 5. Conclusions

Our investigation demonstrated that, although many telemedicine services are already active in Italy, a large part of the population ignores its availability.

Considering that telemedicine can have a positive impact on quality of care, its use must be promoted among citizens [32], as well as in light of future restriction measures adopted to counteract possible emergencies. Moreover, the study highlighted the need for enhancing the awareness of and confidence in telemedicine of the Italian population. However, as underlined by our results, the creation of a national “network” culture of telemedicine should start from healthcare personnel, who had a fundamental role in promoting its adoption. Therefore, the integration of telemedicine in the core curriculum of health degree courses should be considered.

Although related to the Italian population, these findings may contribute to the characterization of the spread and acceptance of telemedicine worldwide.

## Figures and Tables

**Table 1:** Sociodemographic characteristics of participants (*n* = 1002).

Variable	
Age	
mean ± SD	49.9 ± 14.8
range	18–90 years
median (IQR)	52 (20)
Sex	
*n* (%)	
female	481 (48.0)
male	520 (51.9)
other	1 (0.1)
Area of origin	
*n* (%)	
north	315 (31.4)
center	462 (46.1)
south	225 (22.5)
Sentimental relationship	
*n* (%)	
single	69 (6.9)
engaged, not cohabiting	133 (13.3)
engaged and cohabiting, not married	147 (14.7)
married	357 (35.6)
divorced/separated	204 (20.3)
widowed	92 (9.2)
Children	
*n* (%)	
no	478 (47.7)
yes	524 (52.3)
Educational level	
*n* (%)	
mandatory to high school	541 (54.0)
degree and post-degree	461 (46.0)
Occupational status	
student	123 (12.3)
unemployed	230 (23.0)
employed	573 (57.2)
retired	76 (7.6)
Place of residence	
*n* (%)	
own house	909 (90.7)
other’s house (studying/working offsite)	35 (3.5)
other’s house (as a guest)	37 (3.7)
nursing home	21 (2.1)
Chronic disease	
*n* (%)	
no	520 (51.9)
yes	482 (48.1)

**Table 2:** Answers regarding participants’ knowledge and attitude toward telemedicine services (*n* = 1002).

Question	Respondents *n* (%) ^†^
Have telemedicine services been activated in your region?	
No	124 (12.4)
Yes	456 (45.5)
I don’t know	422 (42.1)
If yes, how did you learn of them?	
My doctor	390 (92.4)
My work	5 (1.2)
Advertising	9 (2.1)
Friends or relatives	18 (4.3)
If yes, have you ever used them?	
No	267 (63.3)
Yes, for myself, autonomously	146 (34.6)
Yes, for myself, with someone’s help	5 (1.2)
Yes, for a relative	4 (0.9)
If you have used these services, are you satisfied?	
No	8 (5.2)
Yes	147 (94.8)
If these services are available in your region,	
but you did not use them, why?	
It would be difficult for me to access	98 (36.7)
It would not be difficult for me, but I don’t need them	25 (9.4)
It would not be difficult for me, but I prefer the traditional services	144 (53.9)
If it would not be difficult for you to use these services	
but you prefer the traditional ones, why?	
Privacy reasons	27 (18.7)
I prefer to meet doctors in person	9 (6.2)
I think that these services can be less effective	108 (75.0)
If these services are not yet available in your region, would you use them?	
No	52 (9.5)
Yes	494 (90.5)

^†^ Percentages are calculated from the number of respondents to each question.

**Table 3:** Answers regarding participants’ knowledge and attitude toward electronic health data records (*n* = 1002).

Question	Respondents *n* (%) ^†^
Have electronic health data records been activated in your region?	
No	169 (16.9)
Yes	166 (16.6)
I don’t know	663 (66.4)
If yes, how did you learn of them?	
My doctor	133 (83.1)
My work	4 (2.5)
Advertising	5 (3.1)
Friends or relatives	18 (11.3)
If yes, have you ever used them?	
No	134 (80.7)
Yes, for myself, autonomously	23 (13.9)
Yes, for myself, with someone’s help	9 (5.4)
If you have used this service, are you satisfied?	
No	2 (6.2)
Yes	30 (93.7)
If this service is available in your region but you did not use it, why? It would be difficult for me to access It would not be difficult for me, but I don’t need it to manage my data It would not be difficult for me, but I prefer to keep my data	3 (2.2) 127 (94.8) 4 (3.0)
If it would not be difficult for you to use this service	
but you prefer to keep your data, why?	
Privacy reasons	0
I prefer to decide which data to share with a doctor	0
I am afraid that data can be lost/the service may be unavailable	4 (100.0)
If this service is not yet available in your region, would you use it?	
No	27 (3.2)
Yes	805 (96.8)

^†^ Percentages are calculated from the number of respondents to each question.

**Table 4:** Comparisons of sociodemographic and health characteristics of participants with positive or negative attitudes toward telemedicine services with corresponding *p*-values from the chi-squared test (*n* = 1002).

Variable	Negative Attitude *n* (%)	Positive Attitude *n* (%)	*p*-Value
Age			
≤52 years	18 (34.6)	294 (55.1)	0.007
>52 years	34 (65.4)	240 (44.9)	
Sex			
female	27 (51.9)	246 (46.1)	0.693
male	25 (48.1)	287 (53.7)	
other	0 (0)	1 (0.2)	
Area of origin			
north	10 (19.2)	85 (15.9)	0.372
center	35 (67.3)	334 (62.5)	
south	7 (13.5)	115 (21.5)	
Sentimental relationship			
engaged	21 (40.4)	181 (33.9)	0.431
not engaged	31 (59.6)	353 (66.1)	
Children			
no	28 (53.8)	251 (47.0)	0.425
yes	24 (46.2)	283 (53.0)	
Educational level			
mandatory to high school	31 (59.6)	288 (53.9)	0.522
degree and post-degree	21 (40.4)	246 (46.1)	
Occupational status			
unemployed	25 (48.1)	234 (43.8)	0.657
employed	27 (51.9)	300 (56.2)	
Place of residence			
own house	49 (94.2)	491 (91.9)	0.753
other	3 (5.8)	43 (8.1)	
Chronic disease			
no	7 (25.9)	40 (14.0)	0.171
yes	20 (74.1)	245 (86.0)	

**Table 5:** Comparisons of sociodemographic and health characteristics of participants with positive or negative attitudes toward electronic health data records with corresponding *p*-values from the chi-squared test (*n* = 1002).

Variable	Negative Attitude *n* (%)	Positive Attitude *n* (%)	*p*-Value
Age			
≤52 years	13 (48.1)	401 (49.1)	0.920
>52 years	14 (51.9)	416 (50.9)	
Sex			
female	14 (51.9)	388 (47.5)	<0.001
male	12 (44.4)	429 (52.5)	
other	1 (3.7)	0 (0)	
Area of origin			
north	0 (0)	220 (26.9)	<0.001
center	13 (48.1)	422 (51.7)	
south	14 (51.9)	175 (21.4)	
Sentimental relationship			
engaged	14 (51.9)	291 (35.6)	0.127
not engaged	13 (48.1)	526 (64.4)	
Children			
no	7 (25.9)	377 (46.1)	0.060
yes	20 (74.1)	440 (53.9)	
Educational level			
mandatory to high school	10 (37.0)	424 (51.9)	0.186
degree and post-degree	17 (63.0)	393 (48.1)	
Occupational status			
unemployed	19 (70.4)	336 (41.1)	0.005
employed	8 (29.6)	481 (58.9)	
Place of residence			
own house	13 (48.1)	757 (92.7)	<0.001
other	14 (51.9)	60 (7.3)	
Chronic disease			
no	2 (8.3)	57 (12.5)	0.771
yes	22 (91.7)	398 (87.5)	

## Data Availability

Data are contained within the article.

## Supplementary Materials

The following supporting information can be downloaded at: https://www.mdpi.com/article/10.3390/healthcare11152157/s1, Questionnaire used in the study.
