# Supplementary material for: Introducing Telemedicine in Italy: Citizens’ Awareness of a New Healthcare Resource
Source: Healthcare (Basel). 2023 Jul 28;11(15):2157. doi: 10.3390/healthcare11152157 (PMC10419285; doi:10.3390/healthcare11152157)
Supplement: Supplementary file 1 [file healthcare-11-02157-s001.zip › healthcare-2480999-supplementary.pdf]

Supplementary File S1. Questionnaire used in the study.

TeleMedicine in Italy – TeMI questionnaire

Presentation of the study

In recent years, also in relation to the boost given by social restrictions due to the COVID-19 pandemic, the development of information technologies has made it possible for healthcare institutions to provide some services electronically (so-called telemedicine). Today in various Italian regions it is possible to consult one's health data on the personal electronic file, or undergo medical visits and consultations through the use of the internet. In order to evaluate the perception of the Italian population towards this innovation, we ask you to answer the questionnaire below. Filling out the questionnaire takes only a few minutes.

Participation in this survey is voluntary and anonymous. Your data will be collected and analyzed with respect for privacy and will not be passed on to third parties. All collected data will be used in aggregate mode and only for scientific purposes. The information provided will not allow the participants to be traced.

By participating in this survey, you confirm that you are over 18 years old, that you have been sufficiently informed about the purposes of the study and that you have acquired the necessary information on the processing of your data in compliance with privacy, pursuant to Legislative Decree 101/2018.

1. SOCIO-DEMOGRAPHIC AND HEALTH INFORMATION

Indicate your age __________

Indicate your sex

Male
Female
Other

Please indicate your region of residence ________________________

Please indicate your marital status/current relationship status

Married
cohabitant
I'm in a relationship with someone I don't live with
Separated/Divorced
Widow
Single (never married, not in a relationship currently)

Do you have children?

Yes

No

What is your educational level?

Mandatory to high school

Degree or post-degree

Please indicate your current occupational status

Student

Unemployed

Employed

Retired

Please indicate where you currently live

My home

Someone else's house (studying/working offsite)

Someone else's house (as a guest)

Nursing home

Do you have any chronic condition (hypertension, diabetes, cancer, etc.)?

Yes

No

2. TELEMEDICINE

Are there active telemedicine services in your region (medical visits, consultations, diagnostic services provided via the internet - excluding only booking and report collection services, and electronic health data records*)?*

Yes

No

I don't know

If yes, how did you find out about it?

Through my doctor

I work in healthcare

Through advertising

Through friends/relatives

If yes, have you ever used it?

No

Yes, for me, by myself
Yes, for me, with someone's help
Yes, for a family member of mine

If you have used these services, are you satisfied?
No
Yes

If telemedicine services are active in your region but you have never used them, why?
I haven't needed them but I think I'd be able to use them
I have difficulty using them (I don't have internet access, I don't know how to do it)
I would have no difficulty using them but I prefer to use these services in person

If you answered "I would have no difficulty using them but I prefer to use these services in person" to the previous question, please indicate why
I don't want my data to end up on the internet
I prefer to talk about my health problems in person
I am afraid that the treatments offered in this way would not be complete/effective

If no telemedicine services are active in your region, would you be inclined to use them if they were activated?
No
Yes

3. ELECTRONIC HEALTH DATA RECORDS
Is the electronic health record offered by the Regional Health Service or by your ASL active in your region?
No
Yes
I don’t know

If yes, how did you find out about it?
Through my doctor
I work in healthcare
Through advertising
Through friends/relatives

If yes, have you ever used it?
No

Yes, for me, independently
Yes, for me, with someone's help
Yes, for a family member of mine

If you have used this service, are you satisfied?
No
Yes

If the electronic health record is active in your region but you have never used them, please indicate why
I haven't needed them but I think I'd be able to use it
I have difficulty using it (I don't have internet access, I don't know how to do it)
I would have no difficulty using it but I prefer to keep my health data

If you answered that you prefer to keep your health data, please indicate why
I don't want my data are placed on the internet
I want to be the one to decide what to let the doctor know
I'm afraid that data can be lost/the electronic health record doesn’t work when I need to consult it

If the electronic health record is not yet available in your region, would be inclined to use it if it is activated?
No
Yes
